# Challenges in developing Geroscience trials

**DOI:** 10.1038/s41467-023-39786-7

**Published:** 2023-08-19

**Authors:** Yves Rolland, Felipe Sierra, Luigi Ferrucci, Nir Barzilai, Rafael De Cabo, Joan Mannick, Anthony Oliva, William Evans, Davide Angioni, Philipe De Souto Barreto, Jeremy Raffin, Bruno Vellas, James L. Kirkland, the G.C.T-TF group, Sandrine Andrieu, Daniel Bacqueville, Heike Bischoff-Ferrari, Guillaume Blivet, Timothy Cash, Ana-Maria Cuervo, Noelie Davezac, Andrea Dimet-Wiley, Alexander Fleming, Friedlander Gérard, Mitzi Gonzales, Sophie Guyonnet, Joshua Hare, Suzanne Hendrix, Christian Jorgensen, Matt Kaeberlein, Mehmood Khan, Stephen Kritchevsky, Aksana Labokha, Olivier Lairez, Stephanie Lederman, Jean Mariani, Lefkos Middleton, John Newman, Angelo Parini, Susan Peschin, Marco Prunotto, Sami Sagol, Suzanne Tomlinson, Georg Terstappen, Jacques Touchon, Cendrine Tourette, Rob Van Maanen, Eric Verdin, Stan Watowich, Lei Zhang, Alex Zhavoronkov

**Affiliations:** 1 Gérontopôle de Toulouse, IHU HealthAge, Institut du Vieillissement, Centre Hospitalo-Universitaire de Toulouse, Toulouse, France; 2 CERPOP UMR 1295, University of Toulouse III, Inserm, UPS, Toulouse, France; 3 Hevolution Foundation, Riyadh, Saudi Arabia; 4 National Institute on Aging, National Institutes of Health, Bethesda, MD USA; 5 Institute for Aging Research, Albert Einstein College of Medicine, Bronx, NY USA; 6 Translational Gerontology Branch, National Institute on Aging, National Institutes of Health, Baltimore, MD USA; 7 Life Biosciences, Boston, MA USA; 8 Longeveron Inc., Miami, FL USA; 9 Department of Nutritional Sciences and Toxicology, University of California, Berkeley, CA USA; 10 Noaber Foundation Professor of Aging Research, Mayo Clinic, Principal Investigator, NIH R33 Translational Geroscience Network, President, American Federation for Aging Research, Rochester, NY USA; 11 Centre de Recherche Pierre Fabre, Toulouse, France; 12 Center on Aging and Mobility, Zurich, Switzerland; 13 REGEnLIFE, Montpellier, France; 14 Rejuveron, Zurich, Switzerland; 15 CRCA—Centre de Recherches sur la Cognition Animale—UMR5169, Toulouse, France; 16 Ridgeline Therapeutics, Houston, TX USA; 17 Kinexum, Tolerion, Charlottesville, VA USA; 18 Université Paris Descartes, Paris, France; 19 University of Texas Health Science, San Antonio, TX USA; 20 Pentara Corporation, Salt Lake City, UT USA; 21 Institut de Médecine Régénérative et Biothérapie, Montpellier, France; 22 University of Washington, Seattle, WA USA; 23 Wake Forest University School of Medicine, Winston-Salem, NC USA; 24 American Federation for Aging Research, New York, NY USA; 25 Biophytis, Paris, France; 26 Imperial College London, London, UK; 27 Buck Institute for Research on Aging, San Francisco, CA USA; 28 Alliance for Aging Research, Washington, WA USA; 29 Roche, Basel, Switzerland; 30 Sagol Neuroscience and Longevity Network, Tel Aviv, Israel; 31 Cambrian Biopharma, New York City, NY USA; 32 CHU Montpellier, Montpellier, France; 33 Buck Institute for Age Research, Novato, CA USA; 34 University of Minnesota Minneapolis, Minneapolis, MN USA; 35 Insilico Medicine, Pak Shek Kok, Hong Kong

**Keywords:** Target validation, Cell death, Drug development, Randomized controlled trials

## Abstract

Geroscience is becoming a major hope for preventing age-related diseases and loss of function by targeting biological mechanisms of aging. This unprecedented paradigm shift requires optimizing the design of future clinical studies related to aging in humans. Researchers will face a number of challenges, including ideal populations to study, which lifestyle and Gerotherapeutic interventions to test initially, selecting key primary and secondary outcomes of such clinical trials, and which age-related biomarkers are most valuable for both selecting interventions and predicting or monitoring clinical responses (“Gerodiagnostics”). This article reports the main results of a Task Force of experts in Geroscience.

## Abstract (web summary)

Geroscience is becoming a major hope for preventing age-related diseases and loss of function by targeting biological mechanisms of aging. This article reports a discussion of a research Task Force on the challenges posed by the clinical research in Geroscience so that future gerotherapeutic clinical trials can be conducted successfully.

## Introduction

For over a century, continued improvements in living conditions, public health policies, and advances in medicine have led to an unprecedented increase in life expectancy across the globe. However, older adults now routinely experience multi-morbidities that are strongly associated with the aging process, including cardiovascular diseases, type II diabetes mellitus, dementias, cancers; sarcopenia, frailty, and functional decline, as well as declines in immune function and other systems^1^. Relative to life expectancy, these can result in a prolonged period of decreased health that can last over a decade. The increases in life expectancy that have outpaced increases in healthspan in some countries are characterized by increased disability, diminished quality of life, and high healthcare costs^2^.

Multiple clinical conditions and pathophysiological processes have long been considered as inescapable and unmodifiable consequences of the aging process. However, these perceptions are changing, in particular with respect to how modifying factors, such as levels of physical activity, diet, social and community disadvantage, and exposure to pollution and other environmental stresses may affect the trajectory of developing age-related disabilities and diseases. Over the past few decades, research focusing on the interplay between the fundamental processes of aging and the biology of co-morbidities has given rise to the concept of Geroscience, the goal of which is to develop new biologically-based therapeutic and preventive approaches that target fundamental aging processes; thus, to decrease age-related multi-morbidities as a group and improve healthspan^3^. The beneficial effect of using Gerotherapeutic drugs to modulate the fundamental molecular, cellular, and/or genetic mechanisms of aging has been demonstrated in animal models, and offers exciting preventive and even curative therapeutic translational opportunities in humans^4–9^. However, Geroscience trials face numerous methodological challenges in their study design regarding demonstrating clinical effectiveness successfully in humans^10–12^. One critical challenge is that the usual design of therapeutic clinical trials is centered on disease-specific diagnosis and physiopathology, whereas Geroscience trials aim to target mechanisms of aging in order to delay or prevent the onset or reduce the progression of multiple age-related diseases, geriatric syndromes, and potentially alleviate or treat such conditions.

We present here the recommendations by the Geroscience Clinical Trial International Task Force, which met on March 24 and 25, 2022 in Toulouse, France. Design methodologies of future randomized controlled trials in the field of Geroscience were considered, including the most appropriate target populations and key clinically relevant primary, secondary, and exploratory endpoints.

## What are potential outcomes of Geroscience clinical trials?

Indications for treatment are defined by governmental agencies, such as the Food and Drug Agency (FDA) or European Medicine Agency (EMA) as conditions, usually a disease or manifestation of disease—which affect the life quality and functioning of an individual in the opinion of patients, physicians, and experts in the field (https://www.fda.gov/drugs/development-approval-process-drugs). As such, it is unlikely that regulatory agencies will recognize aging as a treatable indication. Thus, defining approvable indication(s) for Geroscience compounds currently under development is an essential task.

The EMA and the FDA have high concordance (91–98%) in decisions on marketing approvals^13^, but the arrival of gerotherapeutic drugs will challenge both agencies to define the terms of marketing approval in the context of Geroscience. Being an emergent discipline, Geroscience will challenge some of the established protocols for fast approval of new drugs and biomarkers needed to meet the challenges of an aging society. Although some differences may exist between FDA and EMA, these agencies have been dialoguing and cooperating in the past two decades in order to align Europe and the US on decisions on market authorizations^13^. In general, FDA or EMA approve drugs for treating diseases; however, aging by itself is not currently considered as a “disease”, but as the major risk factor for multiple morbidities. Basic scientists, clinicians, and drug agency officials already interact so that the concept behind Geroscience is understood and shared. A scale for evaluating FDA-approved drugs for their Gerotherapeutic potential has been proposed^4^. In this context, it is important to highlight that the design of the TAME (Targeting Aging with MEtformin) trial has been approved by the FDA; TAME aims to delay mortality and the onset of several age-related diseases (e.g., myocardial infarction, stroke, cancer, dementia) and conditions (e.g., major decline in mobility or cognitive function) rather than targeting a single disease^14^. The TAME trial may serve as a proof of concept that proves to the medical agencies that aging can be a therapeutic indication in itself. This result would favor conditions for defining new marketing approval, type of approval, and approved indication for new or already approved drugs and will be incentive for pharmaceutical companies to invest in research on Geroscience. Diet and physical exercise are prevention strategies targeting multiple age-related conditions in humans that are already recognized by all international recommendations for healthy aging. It is demonstrated that both diet and physical exercise influence the hallmarks of aging. Other lifestyle modifications such as the use of probiotics could also be relevant and safe and various drugs, safe, and already on the market, such as metformin or SGLT − 2 inhibitors could be considered to prevent the degradation of the hallmarks of aging.

Experts agree that chronic pathologies that become prevalent in middle-age and older adulthood (i.e. age-related diseases such as type II diabetes mellitus, arthritis, osteoporosis, heart failure, dementias, cancers) share, at their root causes, metabolic, genetic, and cellular dysfunction collectively termed the “hallmarks of aging”^15^. These hallmarks of aging are also thought to underlie various geriatric conditions such as frailty, which is characterized by declines in the domains of intrinsic capacity, including mobility and cognition^12^. A Geroscience trial can be defined by its targeting one or more hallmarks of aging. By acting directly on fundamental aging mechanisms, Geroscience therapeutic candidates are intended to postpone occurrence of multiple age-related diseases, target a single disease, maintain/improve function(s) (Table 1), or improve healthspan and survival (Table 2). Below, the panel of on-going trials presented during the task force meeting illustrates the heterogeneity of study outcomes across the field of Geroscience. Some trials focus on the onset of clusters of diseases (TAME study), some target specific age-related diseases (such as Alzheimer’s disease, AD), while others target very prevalent age-related physical/biological conditions (such as loss of mobility, delirium, or immuno-senescence in frail subjects, or loss of intrinsic capacity with advancing age). Outcomes using composite scores are not without limitations and permissive effects. An intervention study could achieve its main objective on a composite outcome by having efficacy on only one of the items and thus find a broad indication even though the molecule is only effective on a single biological mechanism of a disease and not on the biological mechanisms of aging. The rating scale proposed by ref. ^4^ should reduce this potential bias.

**Table 1:** Outcomes

Outcome	Example of clinical outcome
Diseases	Incidence of new age-related disease, Cardio-vascular events, Infectious disease, Dementia, Sarcopenia, Osteoporosis, unexplained anemia of aging. Progression rate of the diseases
Geriatric syndrome	Delirium, Falls, Frailty, Incontinence, Undernutrition (anorexia of aging)
Physical and biological function	Disability, Physical performance (SPPB, walking speed), Decline of Intrinsic Capacity and its domains (mobility, mood, vitality, hearing, vision, cognition), Immunity
Healthspan and mortality	See Table 3: Summary of studies investigating the effects of candidate Gerotherapeutic drugs on healthspan and mortality in human (Adapted from Kulkarni et al., 2022)

**Table 2:** Target population

Target population in Geroscience trial	Definition	Potential indications
Subjects at risk of age-related diseases	Age-related diseases: Defined as disease with increased incidence in old age and generated by chronic impairment of the hallmark of aging	Atherosclerosis, cardio-vascular disease, cancer, osteo-arthritis, osteoporosis, type 2 diabetes, hypertension, cataract, Alzheimer’s disease, pre-eclampsia, others
Subjects with accelerated aging-like states	Accelerated aging disease: Genetic disorder that result in accelerated aging-like states	Down syndrome, progeria, Werner syndrome
Subject facing an accelerated aging-like condition	Accelerated aging condition: Clinical condition that results in accelerated phenotypic or biological aging	Immobilization, radiation, chemotherapy, exposure to toxins (tobacco, alcohol…), surgery, cardiopulmonary bypass surgery, hypoxia, prematurity, space flight, others
Subjects at risk of geriatric syndromes	Geriatric syndrome: Group of age-related acute or chronic common health condition that are associated with poor resilience	Delirium, fall, functional limitation, undernutrition, frailty, sarcopenia, cognitive impairment, depression, others
Subjects with (or at risk of) declining Intrinsic capacity (IC)	Intrinsic capacity: Concept that represents the composite of all physical and mental capacities	Older adult with abnormal IC domains (mobility, sensory, mood, cognition, vitality) as defined by WHO

### Geroscience trial to collectively prevent age-related diseases

The TAME study aims to demonstrate that by targeting the cellular hallmarks of aging in humans, the onset of several age-related diseases may be delayed. TAME holds the potential to be the first study that could lead to considering aging, or at least multimorbidity, as a therapeutic indication^14^. TAME aims at investigating the effect of a molecule already approved by the FDA and can be considered as a model for the design of future studies evaluating other drugs that are repurposed with an indication of targeting multimorbidity, aging, or age-related dysfunction^4^. Metformin is a generic drug used for its ability to reduce hepatic insulin resistance and hyperglycemia in patients with type 2 diabetes. Metformin has also been demonstrated to prevent the onset of type 2 diabetes mellitus in those with impaired glucose tolerance. Recently, basic research has demonstrated its involvement in many biological pathways of aging and the prevention of age-related diseases^14,16^. Metformin has a pleiotropic mode of action including suppression of inflammation, activation of nutrient sensor AMPK, inhibition of mitochondrial functioning, lowering adipocyte senescence with reduction of SASP, induction of autophagy and favorable shifts in the gut microbiota^17^. Epidemiological studies have indicated that metformin is associated with a reduced incidence of multiple age-related diseases, including cancers and Alzheimer’s disease^14^, as well as all-cause mortality, effects which have been observed not only in individuals diagnosed with diabetes, but also in non-diabetics, thus supporting the hypothesis that metformin can have geroprotective effects^18,19^. TAME will be a six-year randomized controlled trial, conducted in 14 research institutes in the United States, studying the effects of metformin on the incidence of chronic diseases in 3000 subjects. Participants must be between 65 and 80 years old and either have a walking speed of 0.4 to 1 meter per second or have an age-related disease such as cardiovascular disease, cancers, or Mild Cognitive Impairment (MCI) at the start of the study. The primary endpoint of TAME is the time to incidence of any of 5 major age-related conditions (myocardial infarction, stroke, cancer, heart failure, mild cognitive impairment (MCI)/dementia), or death. The hypothesis is that by targeting one or more hallmarks of aging, metformin will delay the development and severity of chronic diseases.

### Geroscience trials to prevent functional limitations and geriatric syndromes

Another clinical trial related to the Geroscience hypothesis involved intervention the geriatric syndrome, frailty^20^, using bone marrow-derived allogeneic Mesenchymal Stem Cells (MSCs, branded as Lomecel-B). The depletion of stem cells compromises tissue regeneration and repair and is the main rationale for stem cell-based replacement strategy. Allogeneic MSCs offer promise as a Gerotherapeutic drug candidate through multiple mechanisms of action that can address multiple mechanism of aging. Among these are anti-inflammatory, anti-fibrotic, and pro-vascular activities, and ability to stimulate intrinsic and pro-regenerative and repair mechanisms^21,22^. The clinical-stage biotechnology company, Longeveron, *Inc*., reported the results of its multicenter phase IIb randomized clinical trial involving 148 participants aged 70 to 85 with a mild to moderate frailty score (Canadian Study on Health & Aging [CSHA] Clinical Frailty Scale score 5–6), a walking distance of 200 to 400 meters on the 6-min walk test (6MWT), and a low-level inflammatory state (defined by serum tumor necrosis factor TNF-α ≥ 2.5 pg/mL). The study evaluated the effect of 4 doses of MSCs (single intravenous infusion of 25, 50, 100, or 200 million [M] cell) vs. placebo. The primary objectives were (i) to show a difference in the 6MWT between the treatment groups *vs*. the placebo group at 6 months and (ii) to establish the potential dose-response effect of MSCs on the 6MWT. The authors reported a statistically significant dose-response effect with a gain in walking distance of around 63 meters at an MSC dose of 200 M cells by 9 months after a single infusion. This physical performance improvement is greater than the distance of 32 meters considered clinically relevant in chronic heart failure patients^23^ or the 19 to 22 meters of improvement deemed clinically relevant in elderly subjects living in the community^24^. This study also reported a dose-dependent decrease in soluble Tie2 (sTie2), a receptor tyrosine kinase expressed on the surface of endothelial cells, consistent with pro-vascular activity. While these promising results support MSCs as a gerotherapeutic candidate, the ability to simultaneously address multiple mechanisms of aging presents a unique challenge in drug development that will need to be addressed in follow-up studies. In particular, developing mechanistic-related biomarkers of efficacy may be challenging given the number of potential targets involved, and may ultimately require a composite biomarker approach to assess effect and effect duration to guide follow-up treatment. Further complicating this is the fact that principal targets may not be uniform across all patients, e.g., patients with age-related frailty versus disease-related frailty may require unique (and again, possibly composite) biomarkers for effect.

### Geroscience trials to treat or prevent specific age-related diseases

Cellular senescence is a complex cell fate that becomes more frequent with age and occurs in the context of inducers, such as reactive oxygen species, telomere attrition, deoxyribonucleic acid (DNA) damage, or oncogene activation. Cellular senescence can entail to changes in expression of multiple molecular mediators, such as p16^INK4a^ or p21^CIP1/WAF1^, that induce essentially permanent cell cycle arrest, resistance to apoptosis; metabolic changes, such as β-galactosidase accumulation in lysosomes; and cell morphological changes. Most senescent cells develop a senescence-associated secretory phenotype (SASP). The SASP varies depending on the cell type that became senescent, how senescence was induced, how long the cell has been senescent, and its microenvironment. The SASP can entail secretion of growth factors, chemokines, metalloproteases (MMPs), cytokines, non-coding nucleotides (e.g., microRNA’s), and other bioactive molecules (e.g., prostanoids, bradykines, ceramides, ROS), which contribute to organ dysfunction, including dysfunction of the brain^25–28^. Neurons, but also glial and brain endothelial cells, have been reported to develop a senescent phenotype^29–32^. Interestingly, several basic and animal research studies support the idea that lower senescent cell abundance is linked to improved healthspan^6,33–36^, including neurodegenerative disorders^37^. Musi et al. reported in mice that senolytic treatment (biweekly administration of Dasatinib and Quercetin for 12-weeks) reduced brain atrophy, decreased white matter pathology, and rescued aberrant cerebral blood flow^37^. These findings support the idea that reducing senescent cell burden in the brain may reduce neurodegeneration in humans^38^ and pave the way to new therapeutic interventions, such as drugs targeting senescent cell anti-apoptotic pathways, senolytics^39,40^. Directly testing the impact of senescence-targeting therapies in a neurodegenerative state, Mitzi Gonzales, et al. have recently reported the first results of the SToMP-AD trial, a pilot clinical trial of senolytic treatment in early-stage AD^41^. Results show that the combination of Dasatinib and Quercetin is safe, penetrates the central nervous system (CNS), and well-tolerated in humans. This pilot study has provided evidence for proceeding with a multisite Phase II clinical trial to evaluate the safety and feasibility of senolytic therapy in AD.

Longeveron also reported results from its Phase 1 Trial for subjects with Alzheimer’s Disease^42^ at the meeting. Thirty-three participants were enrolled who were between 50 and 80 years old with mild AD. Participants were randomized into three groups, receiving a single intravenous infusion of 20 M allogenic MSCs, 100 M allogenic MSC’s or placebo, and were followed-up for 26 weeks post-infusion. Safety was the primary endpoint. No adverse events (AEs) or serious AE (SAEs) were attributed to the product, thus the safety stopping rule was not triggered (meeting the primary endpoint). With the caveat that the trial was not powered for efficacy, statistically significant improvements were observed in vascular endothelial growth factors (VEGF), and inflammation-related biomarkers (Il-4, Il-6, sIl-2α, Il-10, Il-12), and imaging outcomes (left hippocampus volume). In this case, a potential composite biomarker for effect may be plausible that encompasses both fluid-based and imaging readouts. In addition, cognitive decline (measured by the Mini-Mental State Exam) progressed more slowly in the 20 M MSCs arm compared to placebo, although these findings must be considered with caution given the small sample size.

The above paragraph illustrates the point for Alzheimer disease, a very prevalent condition during aging, but there is also a growing number of Geroscience trials designed to treat or prevent specific age-related diseases. For example, Guanabenz, an alpha-2 adrenergic agonist used to treat hypertension, also targets the loss of proteostasis and has been approved by FDA in patients with amyotrophic lateral sclerosis^43^. In a phase 1 clinical trial, oral Nicotinamide Riboside that targets disabled macroautophagy in Parkinson’s disease patients has been reported to improve clinical symptoms and reduced inflammatory markers in the cerebrospinal fluid and blood^44^. In an open-label phase 2/3 randomized clinical trial, elamipretide, that targets mitochondrial dysfunction, has been reported to improve physical performance and muscle strength on Barth syndrome^45^. In a small sample size phase 1 clinical trial Justice, et al. have reported that Dasatinib + Quercetin, a senolytic drug combination that targets cellular senescence, improved physical performance and reduce inflammatory markers in patients with pulmonary fibrosis^46^. These senolytics have also been reported to reduce the inflammatory profile of patients with diabetic kidney disease^47^. Data from the CANTOS study suggests that a treatment targeting the interleukin-1β innate immune pathway with Canakinumab, aimed to reduce chronic inflammation, reduces the incidence of and mortality from lung cancer. In this Phase 3 clinical trial, 10,061 patients with atherosclerosis who had a myocardial infarction and without previous cancer and high C-reactive protein (hsCRP) levels at baseline were randomized with different doses of Canakinumab or placebo. Incident lung cancer was significantly less frequent in the canakinumab group^48^. Overall, these data related to interventions targeting the mechanisms of aging in the context of human diseases suggest that gerotherapeutic drugs could improve the prognosis or even prevent these diseases, and extend healthspan and lifespan. The multiple ongoing studies will certainly bring new data to the field of gerotherapeutic drugs in the short-to-medium term.

### Geroscience trials targeting Intrinsic Capacity (IC)

Recently the World Health Organization (WHO) introduced the concept of age-related decline in IC to ICD-11, defining IC as the composite of all physical and mental capacities that a person can draw on, including biological reserve (https://www.thelancet.com/journals/lanhl/article/PIIS2666-7568(22)00102-7/fulltext10.1016/S2666-7568(22)00102-7). This paves the way for a possible drug approval to treat IC from the EMA and FDA. The construct of IC has been operationalized through assessing five major functional domains: cognition, psychological, locomotion, vitality, and sensory function^49^. IC has been proposed as a metric to assess function in older adults and could prove to be a relevant primary outcome for Geroscience drug trials. Previous work in AD clinical research led to the development of CDR sum boxes (a composite cognitive score) which has recently been recommended by the FDA for Alzheimer’s prevention trials^14^. A similar composite score model could be developed for IC to bring it into use as an endpoint in clinical trials. For the WHO, healthy aging is defined by the ability to maintain function and to continue to be and to do what we value^50^. By manipulating biological mechanisms of aging, Geroscience innovations may help to slow down the loss of IC and to maintain healthy aging. Maintaining function by increasing or preventing the decline in IC associated with aging as well as preventing the onset of frailty in older adults is the main objective of the WHO Integrated Care for Older People (ICOPE) initiative^51^. ICOPE’s goal is that care for older people worldwide maintains and restores intrinsic capacity and functional capacity and help to promote healthy aging. This preventive approach is already implemented in Occitanie (France)^52,53^ with more than 20,000 community-dwelling participants followed digitally. The key preventive strategies for these older adults currently emphasize healthy lifestyle behaviors (e.g., physical activity, nutrition); new strategies may be added or substituted when specific drugs become available to maintain their IC.

An IC score for clinical research could be used to monitor IC trajectories over time and to predict disability in a manner similar to that of the cognitive composite score for AD. The composite IC measure will have to be predictive of functional ability outcomes (e.g., care dependency) and sensitive to changes over time compared to single measures of function. Depending on its reliability, this might help reduce sample sizes for clinical trials.

Geroscience-based interventions, Gerotherapeutics, appear to delay, prevent, alleviate, or treat not only specific clinical disorders in older adults, but can also impact a broad range of biological processes and multiple diseases and disorders. For example, the process of immune dysfunction that occurs with age (so-called immunosenescence) is closely related to infections, autoimmune diseases, and malignant tumors. It has been reported that upregulation of interferon-induced antiviral responses in older adults with a low dose of an mTOR inhibitor may substantially reduce the severity of viral respiratory tract infections in older adults^54^. Targeting immune function is a major area of clinical study in Geroscience.

### Studies investigating the effect(s) of candidate Gerotherapeutic drugs on healthspan and mortality in humans

Mortality and longevity are outcomes that are difficult to apply in randomized controlled trials because they require long-term follow-up and large sample sizes. A study with the sole objective of lifespan extension would take decades and would be very expensive. Therefore, most clinical projects in progress employ composite outcomes monitoring for the development of diseases in addition to death. Recent synopsis by ref. ^4^ identified studies (in rodents or human) performed on drugs already approved by the FDA which might have geroprotective effects, particularly those potentially improving healthspan and extending lifespan. The authors propose a standardized process for evaluating FDA-approved medications for their gerotherapeutic potential. The process proposed is a 12-point rating scale assigning points based on results of robust preclinical and clinical trials for each drug. Six points are related to the results of pre-clinical trials and are attributed when attenuation of the biological mechanisms of aging is achieved or when the extension of lifespan is demonstrated. The six remaining points of the rating scale are related to clinical trials and consider the effects of the drug beyond the targeted disease (the drug has effects on a disease that it was not intended to treat) and are attributed on the reduction of overall mortality or mortality unrelated to the targeted disease. Table 3 gives outcomes of these studies investigating the effects of candidate gerotherapeutic drugs on healthspan and mortality in humans.

**Table 3:** Summary of studies investigating the effects of candidate Gerotherapeutic drugs on healthspan and mortality in human. (Adapted from Kulkarni et al., 2022)

Gerotherapeutics	References	Primary outcome(s)
SGTL-2 inhibitors	Packer et al.^78^	A composite of cardiovascular death or hospitalization for worsening heart failure.
SGTL-2 inhibitors	Perkovic et al.^79^	A composite of end-stage kidney disease (dialysis, transplantation, or a sustained estimated GFR of <15 ml per minute per 1.73 m2), a doubling of the serum creatinine level, or death from renal or cardiovascular causes.
SGTL-2 inhibitors	Heerspink et al.^80^	A composite of a sustained decline in the estimated glomerular filtration rate (GFR) of at least 50%, end-stage kidney disease, or death from renal or cardiovascular causes.
SGTL-2 inhibitors	Neal et al.^81^	A composite of death from cardiovascular causes, nonfatal myocardial infarction, or nonfatal stroke.
SGTL-2 inhibitors	Zinman et al.^82^	A composite of death from cardiovascular causes, nonfatal myocardial infarction, or nonfatal stroke.
SGTL-2 inhibitors	McMurray et al.^83^	A composite of worsening heart failure (hospitalization or an urgent visit resulting in intravenous therapy for heart failure) or cardiovascular death.
Metformin	Knowler et al.^84^	Diagnosis of diabetes.
Metformin	UKPDS Group^85^	Any diabetes-related clinical endpoint (sudden death, death from hyperglycemia or hypoglycemia, fatal or non-fatal myocardial infarction, angina, heart failure, stroke, renal failure, amputation [of at least one digit], vitreous hemorrhage, retinopathy requiring photocoagulation, blindness in one eye, or cataract extraction); diabetes-related death (death from myocardial infarction, stroke, peripheral vascular disease, renal disease, hypoglycemia, or hyperglycemia, and sudden death); and all-cause mortality.
Metformin	Hong et al.^86^	A composite of cardiovascular events, death from a cardiovascular cause, and death from any cause.
Metformin	Luchsinger et al.^87^	The co-primary clinical outcomes were changes from baseline to 12 months in total recall of the Selective Reminding Test (SRT) and the score of the Alzheimer’s Disease Assessment Scale-cognitive subscale (ADAS-cog). The secondary outcome was change in relative glucose uptake in the posterior cingulate-precuneus in brain fluorodeoxyglucose positron emission tomography.
Metformin	Cryer et al.^88^	The primary end point was the incidence of serious adverse events (SAEs), death, and hospitalization.
Acarbose	Holman et al.^89^	To assess whether acarbose could reduce the frequency of cardiovascular events in Chinese patients with established coronary heart disease and impaired glucose tolerance, and whether the incidence of type 2 diabetes could be reduced.
Acarbose	Chiasson et al.^90^	The primary endpoint was development of diabetes on the basis of a yearly oral glucose tolerance test (OGTT).
Acarbose	Chiasson et al.^91^	To evaluate the effect of decreasing postprandial hyperglycemia with acarbose, an alpha-glucosidase inhibitor, on the risk of cardiovascular disease and hypertension in patients with impaired glucose tolerance (IGT).
Rapamycin/Rapalogs	Mannick et al.^92^	To evaluate whether the mammalian target of rapamycin (mTOR) inhibitor RAD001 ameliorated immunosenescence (the decline in immune function during aging) in elderly volunteers, as assessed by their response to influenza vaccination.
Rapamycin/Rapalogs	Mannick et al.^93^	To determine whether low-dose mTOR inhibitor therapy enhanced immune function and decreased infection rates in 264 elderly subjects given the study drugs for 6 weeks.
Rapamycin/Rapalogs	Kraig et al.^94^	To determine the safety and tolerability of RAPA in older human subjects.
Methylene Blue	Wishick et al.^95^	The primary outcome was change on the Alzheimer’s Disease Assessment Scale-cognitive subscale (ADAS-cog) at 24 weeks relative to baseline severity.
Angiotensin-converting enzyme inhibitor and angiotensin receptor blocker (ACEi/ARB)	NAVIGATOR study group^96^	Three coprimary outcomes: the development of diabetes; an extended composite outcome of death from cardiovascular causes, nonfatal myocardial infarction, nonfatal stroke, hospitalization for heart failure, arterial revascularization, or hospitalization for unstable angina; and a core composite outcome that excluded unstable angina and revascularization.
ACEi/ARB	Lithell et al.^97^	The primary outcome measure was major cardiovascular events, a composite of cardiovascular death, non-fatal stroke and non-fatal myocardial infarction.
ACEi/ARB	Ravid et al.^98^	To evaluate the effect of prolonged ACE inhibition on renal function and albuminuria in patients with type 2 diabetes.
ACEi/ARB	Onder et al.^99^	The aim was to see whether ACE inhibitors also prevent reduction in physical performance and in muscle strength in older women who do not have congestive heart failure (CHF).
ACEi/ARB	Lithell et al.^100^	Primary: major cardiovascular events (cardiovascular mortality, non-fatal stroke or non-fatal myocardial infarction).
Dasatinib + Quercetin	Justice et al.^46^	The primary endpoints were retention rates and completion rates for planned clinical assessments. Secondary endpoints were safety and change in functional and reported health measures. Associations with the senescence-associated secretory phenotype (SASP) were explored.
Dasatinib + Quercetin	Hickson et al.^47^	Senescent cell and macrophage/Langerhans cell markers and circulating SASP factors.
Dasatinib + Quercetin	Martyanov et al.^101^	Primary objectives were safety and pharmacokinetics. Secondary outcomes included clinical assessments, quantitative high-resolution computed tomography (HRCT) of the chest, serum biomarker assays and skin biopsy-based gene expression subset assignments. Clinical response was defined as decrease of >5 or >20% from baseline in the modified Rodnan Skin Score (MRSS).

## What is the target population for Gerotherapeutic clinical trials?

Table 3 summarizes potential target populations for Gerotherapeutics trials. The field of clinical trials in Geroscience research goes far beyond the prevention or treatment of age-related disease. Indeed, the concepts of Geroscience potentially apply to the entire life course and open up therapeutic perspectives beyond geriatric medicine^6^. The fundamental biological mechanisms of aging start early in adulthood or even before, as indicated by changes in muscle, brain, and cardiovascular function that can be observed as young as 30 years of age in healthy individuals^55,56^. Genetic disorders which show early onset of aging phenotypes such as Down syndrome, which begins with aging of the oocyte before conception and which is linked to cellular senescence, may also be a target population and used in Geroscience as models of accelerated ageing and help develop geroprotective molecules^57,58^ Cellular senescence has been associated with adverse pregnancy outcomes and modulating this hallmark of aging with gerotherapeutic drugs to prevent preterm birth is currently being considered^59^. The study of hallmarks of aging during the perinatal period and their postnatal and long-term effects also represents a new research path in maternal-fetal medicine and its consequences during later childhood or in adults such as asthma related to cellular senescence caused by perinatal hyperoxia^60^. It also appears that surviving cancer at a young age is associated with early onset of age-related chronic diseases including AD, diabetes mellitus, osteoporosis, or sarcopenia, probably due to an acceleration of the biological processes of aging^61,62^. Furthermore, certain acute infections such as COVID-19 induce cellular senescence thereby promoting the entry of the virus into healthy cells^26,63^. Clinical trials of Gerotherapeutics are therefore not restricted to the older adult population but may extend from frail older adults to childhood cancer or even astronauts exposed to radiation^6,64^.

A currently unanswered question is the age at which an individual would begin taking a Gerotherapy in order to prevent aged-related diseases. This question is important because, according to the theory of pleiotropic antagonism, certain biological mechanisms improve health early in life but impair health later in life^65^. For example, some observational data suggest that young subjects with a high level of insulin-like growth factor 1 (IGF-1) are protected against chronic disease, while elderly subjects with a high level of IGF-1 have an increased incidence of age-related disease and death^66^. This points to the possibility that Gerotherapeutic drugs such as metformin, senolytics, IGF-1, and others could be beneficial when one is old but deleterious when one is young. To date, nothing justifies their use in healthy young subjects. However, certain conditions leading to accelerated aging, such as obesity, forced inactivity (e.g., bed rest), and chemotherapy could serve as early-stage indications for Gerotherapeutic drugs. The synergistic or antagonistic effect of Gerotherapeutic drugs should also be studied. For example, in the MASTER trial, Walton, et al. hypothesized that metformin might enhance muscle gain during progressive resistance exercise training by reducing the inflammatory response. However, the authors observed a significantly greater gain in muscle mass in the placebo group^67^. Some data also suggest that metformin reduces the beneficial effects of physical training on endurance and strength capacities^68^ but other data suggest that muscle quality is preserved with metformin^16^.

Some Gerotherapeutic drugs, such as metformin, have been safely used for more than 60 years. Other more recently discovered molecules, such as sodium-glucose cotransporter-2 inhibitors (SGLT-2) or bisphosphonates, are already being widely used and are deemed safe^69,70^. However, our knowledge about potential long-term effects of other Gerotherapeutic drugs remains partial and fragmented. Due to the poorly-understood risks for these novel compounds, trials investigating innovative Gerotherapeutic drugs are currently focusing on target populations with more severe diseases for which there is no appropriate treatment, e.g., patients with idiopathic pulmonary fibrosis^46^ or advanced cancers^71,72^. The results of these trials will help indicate whether Gerotherapeutic drugs can not only prevent but also treat diseases, even at an advanced stage.

## Geroscience clinical trial task force discussion

The concept of Geroscience is that, by modulating one or more biological mechanisms of aging, it may be feasible to influence the functional loss/pathophysiology of a multitude of organs and thus prevent various diseases and clinical conditions. Given this concept, a clinical trial focused on outcomes that summarize the pleiotropic effects of a geroprotective molecule and its ability to prevent multi-morbidity would dramatically contribute to individual and public health. While this approach could validate the concept of Geroscience more than studies targeting one single function (e.g., strength or cognition) or a disease (e.g., Alzheimer’s, or osteoarthritis), experts from the group recognized that in practice and as a first step, targeting a single disease or a function is more feasible approach initially and has the potential to more imminently benefit human health. Focusing on single outcomes such as cognition also have an easier regulatory path forward. Designing a disease-centric trial remains the only way to date to gain approval from the FDA or EMA, each of which still adheres to the “one disease, one drug” model. The regulatory constraints required for a new drug to be brought to patients and the extent to which the patients benefit from it must also be taken into consideration when designing a trial. However, targeting a single pathology in a clinical trial is not without risk either. Diagnostic criteria change over time, in particular with the emergence of biomarkers, not-withstanding that most diseases of ageing are of complex etiology, resulting from (still poorly understood) interactions between non-modifiable factors (including age, sex, and genetic predisposition) and modifiable factors, related to environmental and other exposures, lifestyle factors, etc. Moreover, it should be emphasized that some Gerotherapeutic drugs could have a very modest and difficult to demonstrate effect in organs evaluated separately, but have a clinically significant overall effect due to their action on the whole organism, and the alternative also exists that a study using a composite score might fail to capture substantial changes within just one domain if not statistically powered for that endpoint alone. A trial centered on only one function or disease is the current conventional approach but is probably not appropriate for certain molecules such as metformin, for which effects are pleiotropic, acting on multiple organs and through multiple biological mechanisms^73^. Demonstrating an effect of SGTL-2 inhibitors or rapamycin on cognition or heart failure alone would probably be very difficult to demonstrate because this would likely require an unrealistic number of participants in a clinical trial. Defining the therapeutic purpose of a molecule, therefore, needs to be considered on a case-by-case basis. The development of Geroscience research also requires partnership with governmental drug-regulating agencies such as the EMA and FDA so that drug indications evolve and can be validated outside the currently restricted environment.

By definition, the effect of a Gerotherapeutic drug on any given organ may correlate with its effect on other organs given that Gerotherapeutic drugs affect the common underlying mechanisms of aging shared by all organs. Gerotherapeutic drug trials are thus exemplified by trials such as the ones for Lomecel-B^42^ targeting frailty and cognition and the rationale behind the design of the TAME study^14^ to target multiple diseases. A key challenge for current Gerotherapeutic drug studies is to design studies that provide a better understanding of the mechanisms through which a Gerotherapeutic drug acting on one organ can at the same time act on the whole organism, as opposed to attempting to determine which clinical trial design might be best for Gerotherapeutic drugs overall. The group additionally stressed the importance of systematically collecting a dataset of common clinical tests and biological samples, whatever the primary outcome of the study, to facilitate cross-study comparability and foster progress in the understanding of the biology of aging.

A limitation of current Geroscience clinical trials is that most trials are small and carried out over a short period of time. Demonstrating progression in clinical phenotypes, such as the transition from robust to frail, is not feasible with small study population and short follow-up. Therefore, it is important to assay variables that change rapidly over time and predict long-term clinical effects. One option is to use validated biomarkers of aging, as has been done with cholesterol to validate the value of statins in preventing cardiac events. Therefore, the identification of valid and easily measurable biomarkers of biological aging is a fundamental issue. The ease of using new techniques such as unbiased omics approaches should rapidly advance research in the field. Although the search for such biomarkers of aging has been very active, currently there are no consensually accepted biomarkers of aging. Biomarkers of aging capable of predicting the rate of aging would make it possible to stratify the individual risk, to develop prevention strategies, and eventually to monitor the response to the treatments implemented. In a narrative review, Gonçalves, et al.^74^, reported many promising biomarkers of frailty within each key biological determinants of the aging process^15^ but none has demonstrated its superiority. It is therefore more likely that a set of biomarkers (such as a set of components of SASP, GDF15, epigenetic clock, telomerase activity…), rather than a single biomarker, would be the most appropriate approach to measure the rate of aging. Biomarkers are likely to be a way in the future to better define the criteria by which a target population for a given treatment may be identified. The hierarchy of hallmarks of aging as proposed by ref. ^75^ in three categories (primary, antagonistic, and integrative hallmark) is controversial but suggests that some biomarkers of aging could be used preferentially to assess and follow the early aging phase (primary biomarkers). Other biomarkers may represent a response to damage at a later stage of aging or in different clinical conditions (antagonistic biomarkers). Finally, the global rate of aging could be assessed by other biomarkers (integrative biomarkers). Further investigation to identify biomarkers to aid Gerotherapeutic drug study recruitment and define early change are needed. For this, pre-clinical studies play a critical role in building the design of clinical trials. An alternative to identification of such biomarkers to define the study population could be to conduct studies targeting the acute resilience framework, e.g., clinical situations such as disability associated with hospitalization, delirium, or acute loss of muscle mass after surgery. In this context, a Gerotherapeutic drug could have a significant effect over a very short period of time, thus providing the proof-of-concept for new molecules. A caveat is the difficulty in this clinical setting to include and collect data prior to the acute event (hip fracture, infection, etc.), making these trials difficult to conduct. In addition, there is also the difficulty of the potential study population’s heterogeneity in this clinical setting. The underlying hypothesis behind the use of gerotherapeutic drugs is that they act simultaneously on different aging mechanisms. Biomarkers of aging would make it possible to limit the problems of heterogeneity of the population. They could also make it possible to better understand which are the mechanisms of aging that are favorably influenced by the gerotherapeutic drug. Nevertheless, it cannot be ruled out that a composite of many outcomes may challenge identification of mechanisms due to the many mechanisms potentially integrated in the multiple outcomes.

Combinatorial therapy, whether multiple Gerotherapeutic drugs or Gerotherapeutic drugs combined with other drugs—even drugs that failed in phase 3 for lack of clinically significant improvement—should also be investigated. A change in the health paradigm from being focused on the treatment of a single disease to focusing on the development of treatments to address aging mechanisms would pave the way for designing novel therapeutic approaches and trial designs. At present, a large number of on-going research approaches are focused on the development of a new class of pleiotropic molecules collectively called Gerotherapeutic drugs. A collective reflection of patient associations, the pharmaceutical/biotech industry, researchers, and drug agencies must be carried out in parallel with the development of these molecules such that the greatest number of individuals can benefit from these therapeutic advances without delay.

Geroscience brings the hope of an increased life expectancy at birth, and a compression of morbidity in late life. The duty of social justice is to lead everyone to be able to access the innovations brought about by advances in Geroscience^76^. The indication of preventive drugs could concern a very large proportion of the population. If Gerotherapeutic drugs are expensive and only accessible to the richest, its consequences would be to accentuate social inequalities. The life expectancy of the richest is currently higher than that of the poorest mainly due to social and environmental factors. Gerotherapic drugs accessible to the greatest number would reduce this inequity. The reflection around Gerotherapeutic drug’ development and their reimbursement must consider these economic, societal and ethical dimensions.

Inequity currently exists in routine care and clinical research where minority populations, particularly older subjects, and subjects most at risk of experiencing poorer outcomes are often excluded from clinical trials. Future treatments in the field of Geroscience are likely to concern a considerable number of subjects with different clinical profiles. Geroscience clinical trials should ensure access to the innovation for all individuals who can benefit from it; that will contribute to detect differences in responses to interventions according to subgroups and enhance the generalizability of the conclusions.

The dream of eternal youth must not lead to drifts in the sale of molecules whose undesirable effects are not known or the thoughtless initiation of research projects raising ethical questions. The credibility of the field of Geroscience depends on it. Independent organizations, such as the Nuffield Council on Bioethics^77^, can be of assistance in addressing the ethical issues raised by Geroscience and providing informed comments to policy makers on emerging issues^76^.

At present, further research is required to better understand the mechanisms through which Gerotherapeutic drugs produce their benefits, to define the most appropriate outcomes for assessing the effects of Gerotherapeutics, to delineate target populations, and to demonstrate the safety and tolerability of these molecules. The science of aging may need to extend beyond its focus on older adults, and shift to include young individuals and, to do so, it will be necessary to develop sensitive biomarkers which accurately characterize aging trajectories. The field of Geroscience is at its infancy, but it provides a promising basis for the management and prevention of age-related diseases, accelerated aging conditions, and geriatric syndromes with a potentially transformative impact on public health.
